# Ghrelin and Breast Cancer: Emerging Roles in Obesity, Estrogen Regulation, and Cancer

**DOI:** 10.3389/fonc.2016.00265

**Published:** 2017-01-09

**Authors:** CheukMan Cherie Au, John B. Furness, Kristy A. Brown

**Affiliations:** ^1^Metabolism and Cancer Laboratory, Centre for Cancer Research, Hudson Institute of Medical Research, Clayton, VIC, Australia; ^2^Department of Molecular and Translational Sciences, Monash University, Clayton, VIC, Australia; ^3^Department of Anatomy and Neuroscience, University of Melbourne and Florey Institute of Neuroscience and Mental Health, Parkville, VIC, Australia; ^4^Department of Physiology, Monash University, Clayton, VIC, Australia

**Keywords:** obesity, breast cancer, estrogen, aromatase, ghrelin, des-acyl ghrelin

## Abstract

Local and systemic factors have been shown to drive the growth of breast cancer cells in postmenopausal obese women, who have increased risk of estrogen receptor-positive breast cancer. Estrogens, produced locally in the breast fat by the enzyme aromatase, have an important role in promoting cancer cell proliferation. Ghrelin, a 28-amino acid peptide hormone, may also influence cancer growth. This peptide is produced in the stomach and acts centrally to regulate appetite and growth hormone release. Circulating levels of ghrelin, and its unacylated form, des-acyl ghrelin, are almost always inversely correlated with obesity, and these peptide hormones have recently been shown to inhibit adipose tissue aromatase expression. Ghrelin and des-acyl ghrelin have also been shown to be produced by some tumor cells and influence tumor growth. The ghrelin/des-acyl ghrelin–cancer axis is complex, one reason being that tumor cells have been shown to express splice variants of ghrelin, and ghrelin and des-acyl ghrelin might act at receptors other than the cognate ghrelin receptor, growth hormone secretagogue receptor 1a, in tumors. Effects of ghrelin and des-acyl ghrelin on energy homeostasis may also affect tumor development and growth. This review will summarize our current understanding of the role of ghrelin and des-acyl ghrelin in hormone-dependent cancers, breast cancer in particular.

## Obesity, Aromatase, and Postmenopausal Breast Cancer

Estrogens, including 17β-estradiol, play a crucial role in breast cancer. In premenopausal women, estrogens are mainly produced by the ovaries. After menopause when the ovaries stop producing measurable levels of estrogens, the stromal cells of adipose tissue, including the breast, become the main site of estrogen biosynthesis (1). In postmenopausal women, local estrogen production and circulating estrogens, suggested to be mainly adipose derived, are associated with driving the proliferation of breast tumor cells (2–7). Aromatase, encoded by the *CYP19A1* gene, is the enzyme responsible for catalyzing the final and key step in estrogen biosynthesis by converting androgens into estrogens. It is expressed in steroidogenic tissues such as ovarian granulosa cells and the placenta, as well as in non-steroidogenic tissues such as bone and adipose tissue (8). The *CYP19A1* gene is located on chromosome 15q21.2 and spans approximately 123 kb, with 9 coding exons (II–X) and a 93-kb regulatory region. Tissue-specific promoters are used to regulate aromatase expression by their interaction with promoter-specific first exons (9). Each of these promoters ensures a fine-tuned tissue-selective regulation of aromatase expression. For example, in normal breast adipose tissue, the majority of aromatase transcripts are derived from activation of the distal promoter, promoter I.4. This promoter is activated by glucocorticoids in the presence of class 1 cytokines such as interleukin 6, interleukin 11, leukemia inhibitory factor, and oncostatin M (OSM) *via* the Janus kinase-1/signal transducer and activator of transcription 3 pathway (10). Promoter I.4 is also activated by the inflammatory mediator, tumor necrosis factor alpha (TNFα), *via* the mitogen-activated protein kinase-AP1 pathway (10). Interestingly, promoter I.4 is also utilized in bone, where aromatase expression is increased in response to dexamethasone and OSM (11). However, in breast adipose tissue of obese women and those with breast cancer, the majority of aromatase transcripts are derived from the coordinated activation of promoters I.3 and II. A number of obesity-associated factors, including inflammatory mediator PGE_2_ and the adipokine leptin, have been shown to stimulate aromatase expression *via* these promoters (12, 13). More recently, the gut-derived hormone, ghrelin, and its unacylated form, des-acyl ghrelin, were shown to inhibit aromatase expression in adipose stromal cells (14, 15).

## Ghrelin and Des-Acyl Ghrelin

**Figure 1:**
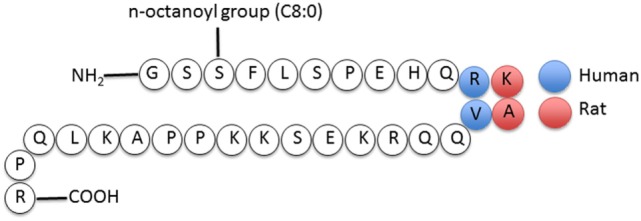
**Structure of human and rat ghrelin (31)**.

**Figure 2:**
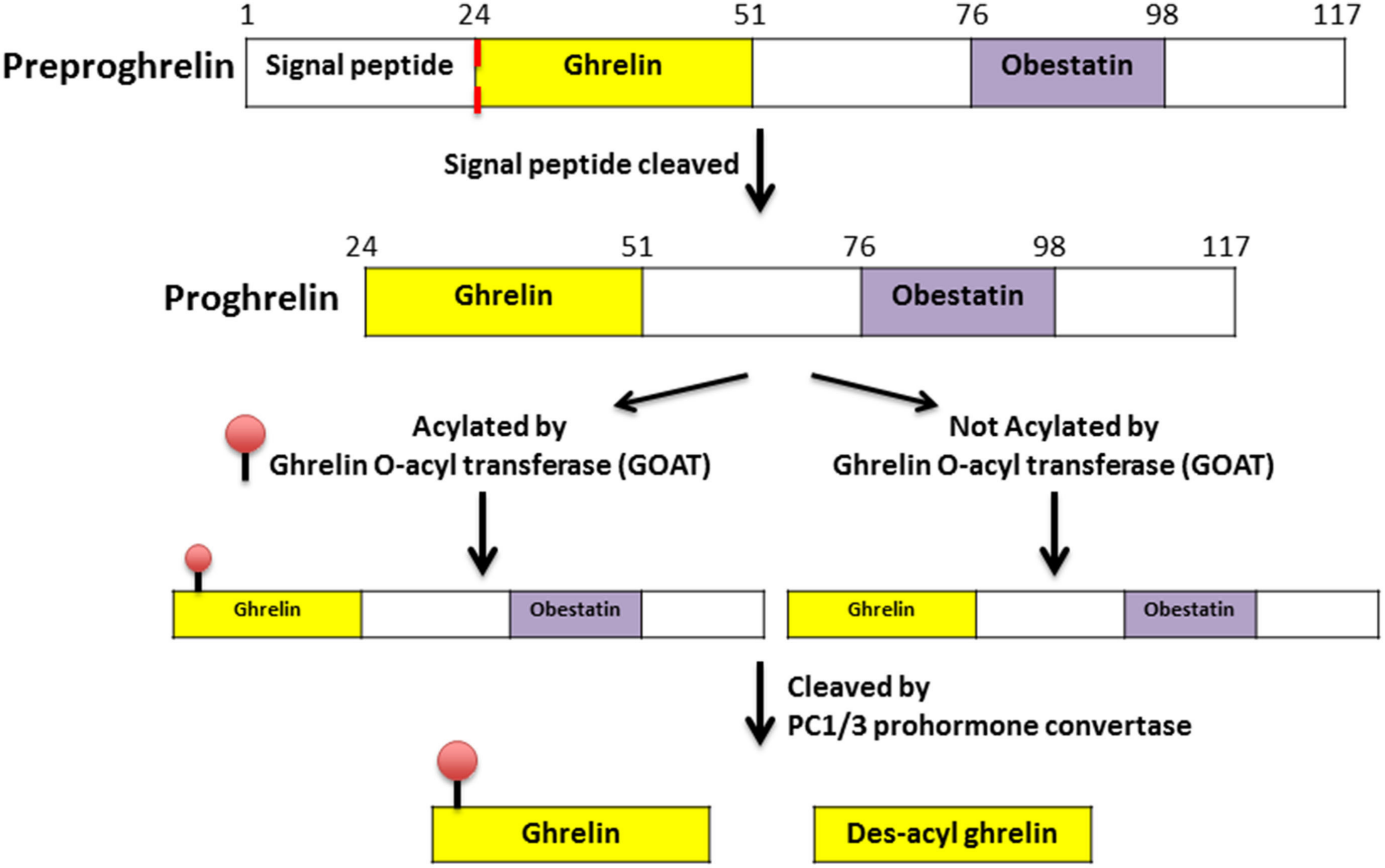
**Processing and acylation of the *GHRL* gene product to yield ghrelin and des-acyl ghrelin (30)**.

Ghrelin, first discovered in the rat stomach in 1999, is the only known peptide hormone to be acylated (16, 17). The human ghrelin peptide is characterized by the level of acylation at serine-3, including non-acylated, octanoylated (C8:0), decanoylated (C10:0), and decenoylated (C10:1) (18). The rat and human ghrelin are generally acylated with an *n*-octanoyl group (C8:0). The structure of human and rat ghrelin differs at two amino acids (Figure 1) (16). In addition to the gut, ghrelin is also produced in small quantities by many tissues including the hypothalamus, pituitary, lung, heart, kidney, testis, pancreas, colon, ileum, jejunum, and duodenum (19–22). The ghrelin gene (*GHRL*) is located on chromosome 3p25-26 and encodes a mature mRNA that is 511 bp long (23–25). Ghrelin mRNA is then translated into preproghrelin, which contains 117 amino acids and can then be acylated and processed into ghrelin and obestatin (Figure 2). Ghrelin-*O*-acyl transferase (GOAT) is the only enzyme that has been molecularly identified to be able to acylate ghrelin (26–28). The ghrelin pro-peptide is cleaved by signal peptidase, prohormoneconvertase 1/3 (PC 1/3) (29), and carboxypeptidase-B like enzyme (18). Whereas the enzymes that cleave the obestatin peptide from the prohormone are still unknown. Preproghrelin can also be cleaved without first being acylated yielding des-acyl ghrelin and obestatin.

Previous studies have demonstrated that ghrelin is the major active form of the hormone acting through the well-characterized ghrelin receptor, growth hormone secretagogue receptor 1a (GHSR1a). GHSR1a is expressed in many tissues such as the pituitary, hypothalamus, stomach, adipose tissue, bone, and prostate, as well as in numerous tumor types such as prostate, breast, and ovarian cancer. Ghrelin binding to GHSR1a leads to activation phospholipase C and generation of inositol triphosphate and diacylglycerol, leading to the stimulation of intracellular calcium release. However, studies have indicated that the mechanism of action of ghrelin in adipose stromal cells is different from the action of ghrelin *via* GHSR1a in the CNS (14, 32). Recent studies have demonstrated that des-acyl ghrelin is also biologically active (33) and likely acts though an unidentified alternative ghrelin receptor (14, 34, 35).

## Roles of Ghrelin and Des-Acyl Ghrelin in Regulating Appetite, Energy Homeostasis, and Obesity

Although it is an orexigenic hormone, levels of ghrelin are inversely correlated with BMI in Caucasians and Pima Indians and reduced in individuals with type II diabetes (36, 37). Moreover, obese individuals have low ghrelin levels before and after meals compared with normal weight individuals (38), a finding that is consistent in obese females (39) and teenagers (40). The role of ghrelin to stimulate growth hormone release also contributes to the regulation of energy homeostasis. Rats receiving subcutaneous ghrelin (200 µg) show stimulated glucose production in the liver, effects that are inhibited by des-acyl ghrelin (41). Consistently, GOAT knockout mice have been shown to have low glucose levels after a low calorie meal compared with ghrelin- or growth hormone-treated mice (33). Different effects of ghrelin and des-acyl ghrelin are largely attributable to differences in receptor usage. Beneficial effects of des-acyl ghrelin on glucose homeostasis have led to the evaluation of AZP531, a cyclic des-acyl ghrelin analog and mimetic, in clinical trials for the treatment of type II diabetes (42). AZP531 has no agonist activity at GHSR1a.

## Ghrelin, Des-Acyl Ghrelin, and Aromatase

The effect of ghrelin and des-acyl ghrelin on aromatase expression in adipose stromal cells was recently examined (14, 15). In isolated adipose stromal cells in culture, ghrelin and des-acyl ghrelin were equipotent (picomolar doses) at suppressing both the basal and PGE_2_-stimulated expression and activity of aromatase. GHSR1a was undetectable in these cells. Ghrelin was found to inhibit cAMP formation, suggesting that ghrelin and des-acyl ghrelin act at a receptor other than GHSR1a, where ghrelin but not des-acyl ghrelin is an agonist. The expression of aromatase in obesity and breast cancer is dependent on cAMP. Hence, decreased levels of ghrelin in obesity provide a possible mechanism for the obesity-associated increase in aromatase within the breast. Obesity is also associated with chronic low grade inflammation and an increase in macrophage-derived inflammatory mediators including TNFα and PGE_2_ (43, 44). Both ghrelin and des-acyl ghrelin suppress macrophage-derived inflammatory factors (15, 45), including TNFα and the expression of COX-2, the rate-limiting enzyme in prostaglandin biosynthesis. Pretreatment of macrophages with des-acyl ghrelin reduced the effect of conditioned media to stimulate aromatase expression in breast adipose stromal cells (15).

## Ghrelin in Cancer

Ghrelin has been implicated in a multitude of cancers including colorectal, pituitary, head and neck, esophageal, liver, gastrointestinal, lung, neuroendocrine, non-Hodgkin lymphoma, pancreatic, thyroid, ovarian, prostate, and breast (46). Ligand-binding assays performed on non-tumoral and neoplastic breast tissue confirmed that the ghrelin analog, hexarelin, was capable of binding to breast cancers, with highest affinity demonstrated for well-differentiated invasive breast carcinomas (47). Interestingly, the ligand was displaced by a number of ghrelin analogs, as well as des-acyl ghrelin, suggesting that ghrelin and des-acyl ghrelin may act on breast cancer cells. This provides further evidence for the presence of alternate ghrelin receptors.

In a study of 53 female patients, including women with benign ovarian tumors and those with ovarian cancer, plasma concentrations of acyl ghrelin were significantly higher compared to the control population, whereas there was no difference between cancer and control groups when total ghrelin plasma levels were compared (48). Due to a lack of scientific evidence for the presence of GHSR1a in human ovaries, the authors suggested that there is likely no direct linkage between ghrelin levels and ovarian cancer development (48). However, now that other receptors for ghrelin are known to exist, this conclusion might be revised. In prostate cancer, a study of 30 patients with benign prostate hyperplasia and 50 controls found no association between the levels of ghrelin and cancer development or progression (49). However, a study by Malendowicz et al. demonstrated that acyl ghrelin and the ratio of acyl ghrelin to total ghrelin were significantly higher in 18 prostate cancer patients compared with 12 benign prostate hyperplasia controls. Total ghrelin plasma levels were similar between prostate cancer and the control group (50).

To date, few studies have examined circulating ghrelin levels in women with breast cancer or with a history of breast cancer (51, 52). However, these focused on effects of therapies, including chemotherapy and isoflavonoids, on circulating ghrelin levels as opposed to relating blood levels with cancer risk. Numerous studies have demonstrated that SNPs in the ghrelin gene are associated with breast cancer risk (53–57), and a study in women with low Native American ancestry also demonstrated that SNPs in the ghrelin gene are associated with breast cancer-specific mortality (58). However, in the absence of functional data, it is difficult to assign causality to these findings.

In some hormone-dependent cancers, ghrelin has been shown to have pro-proliferative effects, whereas in others, it had anti-proliferative effects. Thus, heterogeneity among cancers extends to their responses to ghrelin and des-acyl ghrelin.

## Complexity of the Ghrelin Axis in Cancer

*In vitro* studies have demonstrated that ghrelin can have both proliferative and anti-proliferative effects. Ghrelin stimulates the growth of various cancer cell lines derived from the endometrium (59), prostate (60, 61), and breast (62). Conversely, ghrelin has also been shown to inhibit the growth of small cell lung carcinoma (63), prostate (64, 65), and breast cancer cell lines (47). In the estrogen receptor (ER) + human breast cancer cell line, MCF7, ghrelin at concentrations of 0–1,000 nM had no effect on cell proliferation (62), whereas Cassoni et al. demonstrated that ghrelin, des-acyl ghrelin, as well as a number of ghrelin mimetics, significantly inhibited cell proliferation (47). This effect was also seen in ER + T47D and the triple-negative breast cancer cell line MDA-MB-231, while other groups demonstrated that ghrelin at 10 and 100 nM stimulates MDA-MB-231 cell proliferation (62, 66). Differences in culture conditions or cell lines used may have contributed to these discrepancies. In particular, the addition of 4-(2-aminoethyl) benzene-sulphonyl fluoride hydrochloride (AEBSF) in the study by Jeffery et al. may have prevented the conversion of ghrelin to des-acyl ghrelin, while the effects observed in response to ghrelin in the absence of AEBSF in the Cassoni et al. paper may have in fact been responses to des-acyl ghrelin.

In addition to its well-characterized expression in the stomach, ghrelin is also produced at many other sites, including breast tumors. Breast cancer patients with tumors that express ghrelin have a 2.5 to 3 times lower risk of recurrence or breast cancer-associated death compared to those lacking expression of ghrelin (67). The ghrelin gene can also be alternatively spliced and the transcript variants have been described in breast and prostate cancer (68, 69). In breast, a variant of the ghrelin gene product that includes intron 1 (In1-variant) with a conserved first 12 amino acids and different C-terminus, was detected in tissues where GOAT was expressed and elevated in tumor tissue compared to normal. Although GHSR1a mediates many of the endocrine functions of ghrelin, GHSR1a is absent in several breast cancer cell lines, including MCF7 and MDA-MB-231 cells, and these cells respond to des-acyl ghrelin *in vitro* (47). Moreover, we have recently shown that ghrelin and des-acyl ghrelin inhibit aromatase expression in the absence of GHSR1a, suggesting that ghrelin may act though an unidentified alternative receptor in the breast (14). Another GHSR isoform, GHSR1b, has been identified. It has a truncated, 5-transmembrane, structure and has been proposed to be a non-functional form of GHSR. GHSR1b has been found to be expressed in lung cancer cell lines, including LC319 and PC14 (70), in the epithelium of breast cancer tissues and in breast cancer cell lines, including MDA-MB-231, MCF7, T47D, and MDA-MB-435 (62, 68). It has also been detected in benign prostatic hyperplasia (65), human erythroleukemic cell lines (71), colorectal cancer cells (72), the human adrenal cortex (73), and in benign and malignant adrenocortical tumors (74). Interestingly, GHSR1a and GHSR1b are not expressed in all tissues and tumors where ghrelin is expressed. Therefore, the roles of ghrelin and des-acyl ghrelin differ between cancers. To exploit the potent inhibitory (anti-proliferative) effects that they sometimes exert will require characterization of individual cancers and a personalized approach to treatment with compounds that mimic ghrelin or des-acyl ghrelin’s tumor inhibiting effects.

## Obestatin and Cancer

Obestatin has been implicated in numerous cancers including gastric, ovarian, thyroid, invasive breast, and prostate cancers.

Exogenous obestatin stimulates KATO-III gastric cancer cell proliferation *via* MAPK, ERK1/2-dependent pathways (75). In gastric cancer, obestatin binds to GPR39 leading to the formation of a GPR39/β-arresin/Src complex, which leads to the activation of AKT signaling *via* EGFR transactivation (76). Obestatin in GPR39-bearing gastric cancer cells stimulates epithelial–mesenchymal transition and angiogenesis, as well as affecting morphology, migration, invasion, and proliferation of these cells (77). Furthermore, obestatin causes anti-proliferative effects in TT medullary thyroid carnicinoma cells as well as in pancreatic neuroendocrine tumor cells (78).

With regard to the relationship between circulating peptides and cancer, both obestatin and ghrelin levels were found to be elevated in benign and malignant ovarian cancers (48). No significant associations between circulating obestatin levels and prostate cancer were observed (50).

Expression of obestatin has also been examined in thyroid cancer where levels where found to be elevated in nodular goiter (benign thyroid tumor), but reduced in medullary cancer (malignant thyroid tumor) (79). Conversely, ghrelin levels increased with malignancy. The authors suggested that the ghrelin gene may be alternatively spliced or that ghrelin peptide production may be independently regulated.

In invasive breast cancer and similar to findings relating to ghrelin, the expression of obestatin is weakly correlated with low histological grade, ER positivity, small tumor size, and low proliferative index (67). However, the expression of obestatin did not predict survival.

## Conclusion

Obesity-related breast cancers are largely estrogen dependent. Increased expression of aromatase in obese/inflamed breast adipose tissue contributes to the cancers. In some cases, reduced levels of ghrelin and des-acyl ghrelin may release estrogen biosynthesis and proliferation from inhibition. The ability of ghrelin and des-acyl ghrelin to reduce estrogen production and breast cancer growth may support their use, or ghrelin/des-acyl ghrelin mimetics, as therapeutics for suitably characterized cancers in the future.

## Author Contributions

Conception of article, drafting, and review (CA, JF, and KB).

## Conflict of Interest Statement

The authors declare that the research was conducted in the absence of any commercial or financial relationships that could be construed as a potential conflict of interest.
